# Feasibility of Neuromuscular Electrical Stimulation in a Young Patient With Hematological Malignancy Unable to Perform Active Exercise: A Case Report

**DOI:** 10.7759/cureus.115916

**Published:** 2026-09-07

**Authors:** Shuri Nakao, Shuhei Ishida, Norimasa Egusa, Akihito Noguchi, Yuko Michibata, Sokichi Maniwa, Yasuo Sakai

**Affiliations:** 1 Division of Rehabilitation Medicine, Shimane University Hospital, Izumo, JPN; 2 Department of Rehabilitation Medicine, Shimane University Faculty of Medicine, Izumo, JPN

**Keywords:** exercise intolerance, hematological malignancies, neuromuscular electrical stimulation, physical therapy, thrombocytopenia

## Abstract

Patients with hematological malignancies often have difficulty performing aerobic and resistance exercises because of the disease and the adverse effects from chemotherapy. Neuromuscular electrical stimulation (NMES) may provide an alternative rehabilitation option for patients with cancer who cannot perform conventional exercise therapy. However, the safety and feasibility of NMES in patients with severe thrombocytopenia remain unclear. This case report describes the feasibility of NMES during chemotherapy in a young patient with B-cell acute lymphoblastic leukemia (B-cell ALL), severe thrombocytopenia, and cachexia.

The patient was a woman in her 20s with B-cell ALL and cachexia. Severe fatigue and thrombocytopenia prevented her from performing aerobic or resistance exercises. After consultation with a physiatrist and hematologist, belt-electrode NMES was introduced to maintain and improve muscle strength.

The hematologist approved NMES use regardless of platelet (Plt) count, provided the purpose was to induce muscle contraction. Belt electrodes were applied to the trunk, thighs, and lower legs. NMES was administered at 20 Hz for 20 minutes with a duty cycle of 5 seconds of stimulation followed by 2 seconds of rest. The stimulation intensity was set at the maximum tolerable level that produced visible muscle contractions. Pain, discomfort, and petechial bleeding were monitored during and after NMES. No adverse events, such as bleeding or skin reactions, were observed, even when the Plt count dropped to 19,000/μL. In this case, NMES was feasible and was performed safely in a patient with B-cell ALL who was unable to perform conventional exercise therapy during chemotherapy. This case demonstrates that NMES was feasible and well tolerated under the reported stimulation parameters in one patient with B-cell ALL; confirmation through prospective studies with larger samples is required.

## Introduction

In patients with hematological malignancies, the disease causes muscle weakness, fatigue, and shortness of breath [1]. Additionally, approximately half of patients with cancer experience cachexia [2], which is characterized by decreased skeletal muscle and fat mass [3]. Patients with hematological malignancies often develop cachexia, primarily due to chemotherapy side effects such as nausea, fatigue, and inactivity [4].

A combination of aerobic and resistance exercises has been shown to be effective for patients with hematological malignancies [5]. Exercise therapy improves endurance, muscle strength, and quality of life in patients with hematological malignancies [6]. Despite these benefits, chemotherapy side effects such as neutropenia, anemia, and platelet (Plt) depletion often limit the implementation of exercise therapy [7,8]. Low Plt counts limit exercise therapy to low-intensity activities regardless of physical function [9].

Neuromuscular electrical stimulation (NMES) offers a new rehabilitation option for patients with cancer who have difficulty performing resistance or aerobic exercises [10]. In a previous study, NMES was applied to the upper and lower limbs for 15 minutes, five days a week during chemotherapy for hematological malignancies. This resulted in stable Short Physical Performance Battery (SPPB) scores, with no decrease in post-treatment compared with pre-treatment [11]. However, that study excluded patients unable to perform the 6-minute walk test (6MWT), and the safety and efficacy of NMES in cases with severe thrombocytopenia remain unclear.

This case report describes the feasibility of NMES applied to the entire lower limbs in a patient with B-cell acute lymphoblastic leukemia (ALL) and cachexia who was unable to perform conventional exercise during chemotherapy.

This study was presented as a poster at the 8th Japanese Society of Physical Therapy for Oncology and Lymphedema (JSPTOL), held on November 2, 2025.

## Case presentation

The patient was a woman in her 20s with a height of 145.5 cm, weight of 33.5 kg, and body mass index of 15.8 kg/m^2^. Six months prior to admission, she was treated with antibiotics in Prefecture A for unexplained fever and neutropenia. She was admitted to our hospital on day X for thorough examination and treatment, from her parents' home in Prefecture B. Prior to admission, the patient was independent in activities of daily living. Her occupation was hairdressing, but she resigned during treatment in Prefecture A. Her performance status (PS) at admission was 2 due to fever and fatigue. On day X+2, she was diagnosed with B-cell ALL. Induction remission therapy was initiated on day X+15, with fertility preservation beforehand. Figure 1 shows the course of hospitalization.

**Figure 1:**
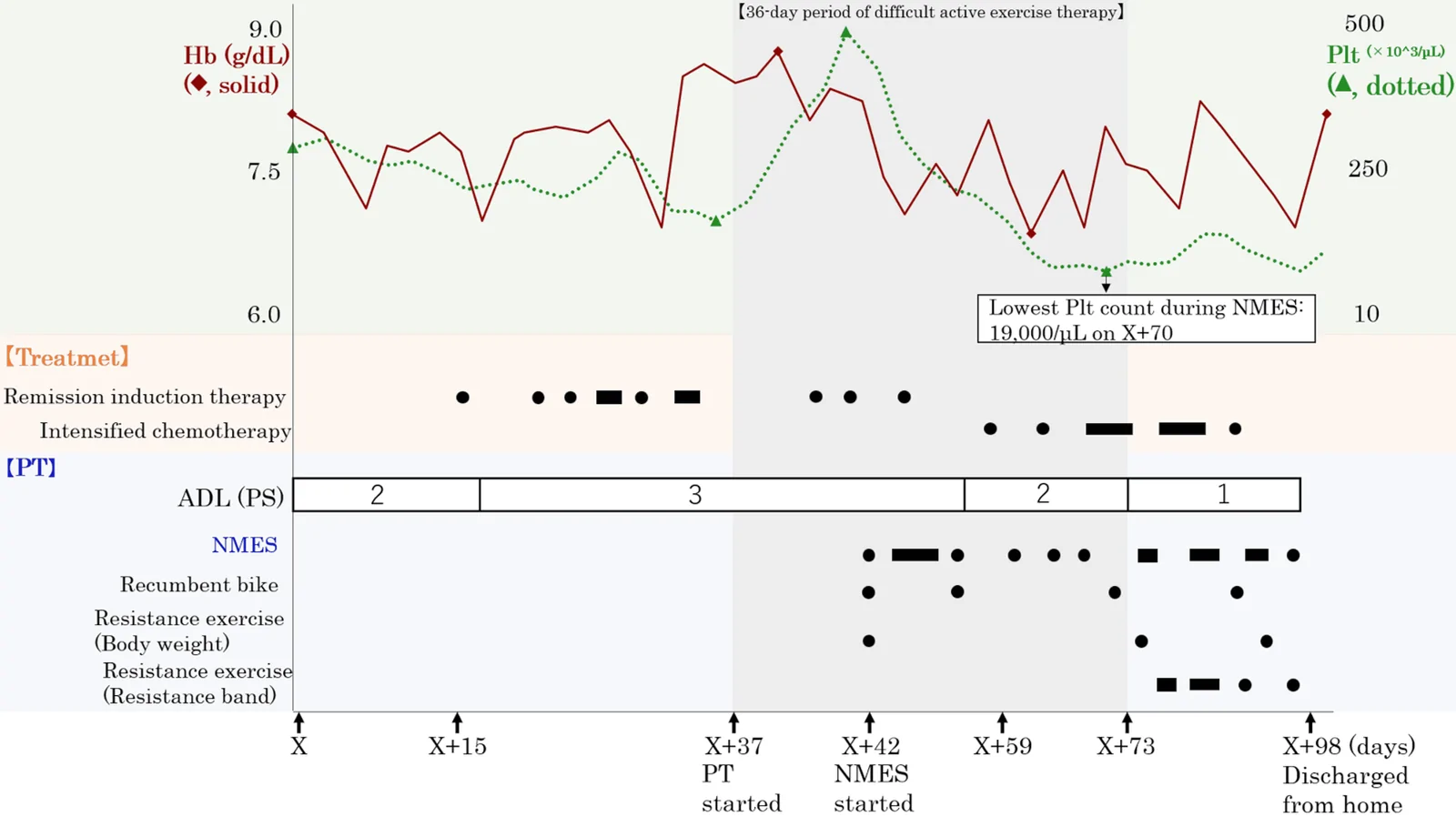
Process Hb, Hemoglobin; Plt, Platelet; PT, Physical Therapy; ADL, Active of Daily of Living, PS; Performance status, NMES; Neuromuscular electrical stimulation Hemoglobin: solid red line with diamond markers (◆) Platelet count: green dotted line with triangle markers (▲) Chemotherapy and NMES: ●, a single treatment session; ■, treatment sessions on consecutive chemotherapy ADL (PS): Performance status over the clinical course X: Day of admission The physical therapy program was tailored to the patient's platelet count, fatigue level, and overall clinical condition. Gray shaded area: period during which conventional active exercise therapy was difficult to perform.

Physical therapy (PT) was initiated on day X+37. On day X+39, a physical function assessment showed the following: grip strength 6.0/5.5 (Rt/Lt) kg, chair stand 5 times (CS5) 25.3 s, 4-m gait 0.48 m/s, 10-m gait 0.52 m/s, and calf circumference (CC) 26.5/26.0 (Rt/Lt) cm (Table 1). Due to pronounced fatigue, the patient was unable to perform the 6MWT. Despite her young age, the patient exhibited poor physical function and fatigue. She had difficulty performing aerobic and resistance exercises, which are known to enhance exercise tolerance and muscle strength in patients with hematological malignancies [5]. This case met the Asian Working Group for Cachexia criteria based on diagnosis, grip strength, and fatigue [12].

**Table 1:** Physical Function X: Day of admission

	X+39 days	X+98 days
Grip strength (kg) (Right)	6.0	14.7
Grip strength (kg) (Left)	5.5	13.6
Chair stand 5 times (s)	25.3	12.2
4 m gait (m/s)	0.48	0.83
10 m gait (m/s)	0.52	0.97
Calf circumference (cm) (Right)	26.5	30.5
Calf circumference (cm) (Left)	26.0	30.0

NMES protocol and feasibility

On day X+42, we introduced NMES for muscle strengthening after consultation with a physiatrist and hematologist. The hematologist approved the use of NMES regardless of Plt count, provided that the objective was muscle contraction. NMES was administered using the belt-electrode method with the General Therapeutic Electrical Stimulation system (G-TES®; Homer Ion Corp., Osaka, Japan). Belt electrodes were attached to the patient's trunk, bilateral thighs, and bilateral lower legs (Figure 2). These belt electrodes target broad muscle groups of the lower extremities, with demonstrated significant activation of the gluteal muscles, quadriceps femoris, sartorius, and hamstrings [13]. NMES was performed at 20 Hz for 20 minutes with a duty cycle of 5 seconds of stimulation, followed by 2 seconds of pause [14]. Stimulation intensity was set at the maximum tolerable intensity that induced visible muscle contraction (motor threshold or above). During NMES application, pain and discomfort were assessed based on the patient's tolerance to the stimulation. The skin was visually inspected for petechial bleeding immediately after NMES and again at the rehabilitation session on the following day; no standardized skin assessment protocol was used. A recumbent ergometer (AERO BIKE 2100R®; Chuo-Health, Kiyosu, Japan) was introduced to improve exercise tolerance (Figure 1). The target Borg scale was 12-14, with an initial exercise duration of 6 minutes [15]. However, severe fatigue made it difficult to consistently use a recumbent ergometer on some days. In addition, depending on the patient's physical condition, we began training the gluteal muscles, quadriceps, and calf muscles using body weight (Figure 1).

**Figure 2:**
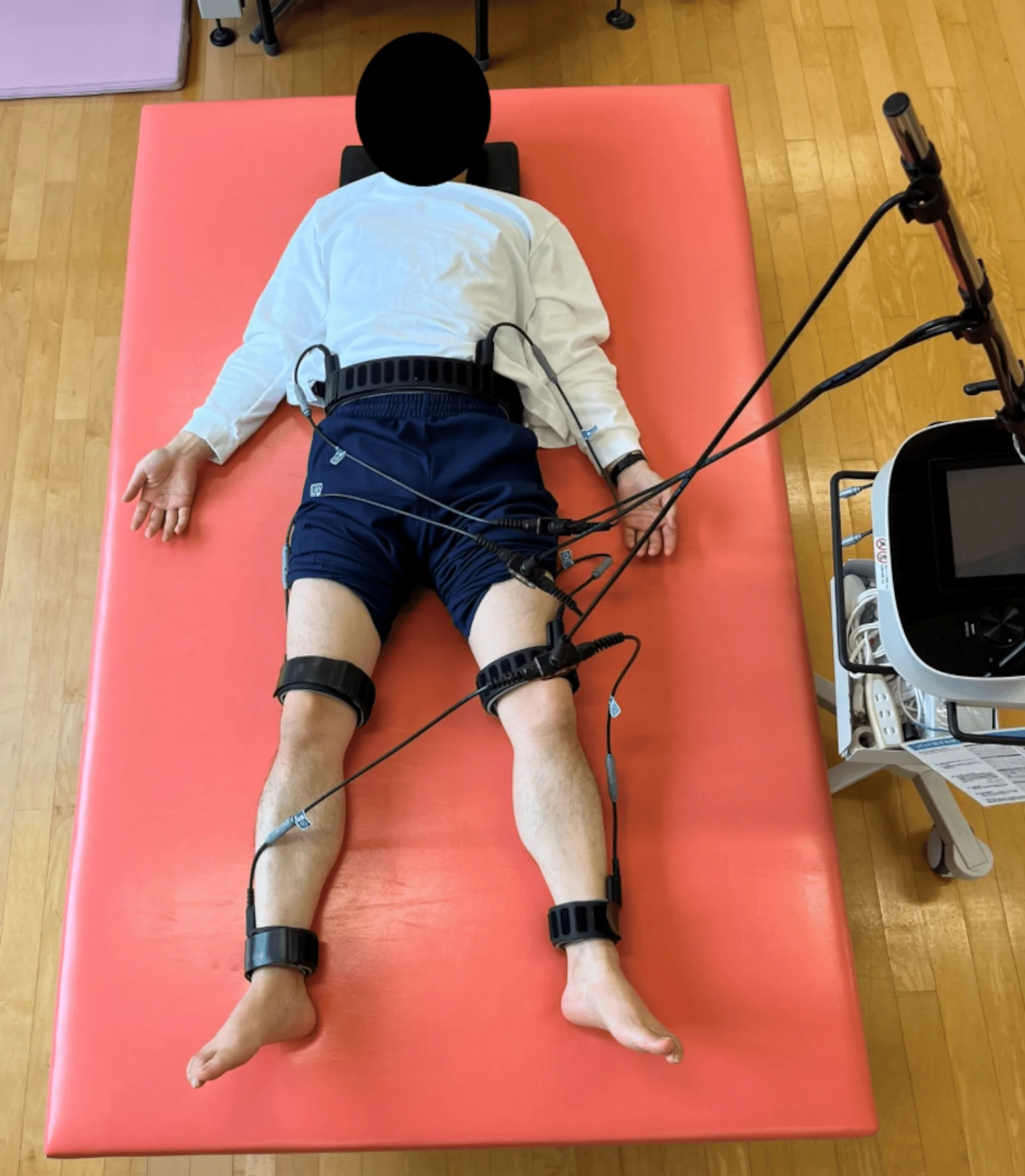
Representative NMES application in a healthy volunteer. NMES; Neuromuscular electrical stimulation NMES was administered using the General Therapeutic Electrical Stimulation belt-electrode system (G-TES®; Homer Ion Corp., Osaka, Japan).

On day X+43, the second course of remission induction therapy was initiated. Severe fatigue persisted due to chemotherapy side effects, and her PS deteriorated to 3. Later, fatigue decreased, allowing her to walk in the ward, and her PS improved to 2.

On day X+70, the patient's PS improved to 1, despite a decrease in the Plt count to 19,000/µL (reference range, 158,000-348,000/µL [16]) (Table 2, Figure 1). Due to the low Plt count, the physiotherapy program was adjusted according to Morishita et al. [9] (Figure 1). Starting on day X+73, resistance-band training targeting the gluteal muscles, quadriceps, and triceps surae was added according to the patient's physical condition (Figure 1). NMES was combined with body weight and resistance-band exercises according to the patient's condition. The flow of physical therapy scheduling and NMES implementation is shown in Figure 3. From day X+42 to X+98, PT was feasible on 33 days, and the patient received PT on 20 of these days. PT was not performed on days when chemotherapy was administered or when the patient requested rest owing to examinations or fatigue. Of the 20 physiotherapy days, NMES was applied for 13 days (65%), whereas the resistance or aerobic exercises could only be used for 2 days (10%) because of fluctuating fatigue. NMES was not performed on seven PT treatment days because of: physical function assessments (2 days), patient inability to tolerate recumbent ergometer or resistance exercise (3 days), petechial bleeding observed before NMES (1 day), and other reasons (1 day) (Figure 3). No NMES sessions were discontinued after initiation due to patient intolerance or clinical instability.

**Table 2:** Weight progression and blood tests BW, Body Weight; Hb, Hemoglobin; Plt, Platelet; Alb, Albumin; TP, Total Protein; N/A, Not Applicable; X: Day of admission Reference ranges are based on the Japanese common reference intervals reported by Ichihara et al. [16].

Laboratory test	Reference range	X+37 days	X+42 days	X+58 days	X+62 days	X+70 days	X+72 days	X+84 days	X+93 days	X+98 days
Hb (g/dL)	11.8–14.8	8.6	8.5	6.4	N/A	6.1	N/A	6.5	6.7	8
Plt(×10^3^/μL)	160–353	133	153	231	N/A	19	N/A	87	12	N/A
Alb (g/dL)	4.1–5.1	3	3.2	3.1	N/A	3.6	N/A	4.2	3.9	3.9
TP (g/dL)	6.6–8.1	5.3	5.7	5.6	N/A	5.9	N/A	6.7	6.4	6.7

**Figure 3:**
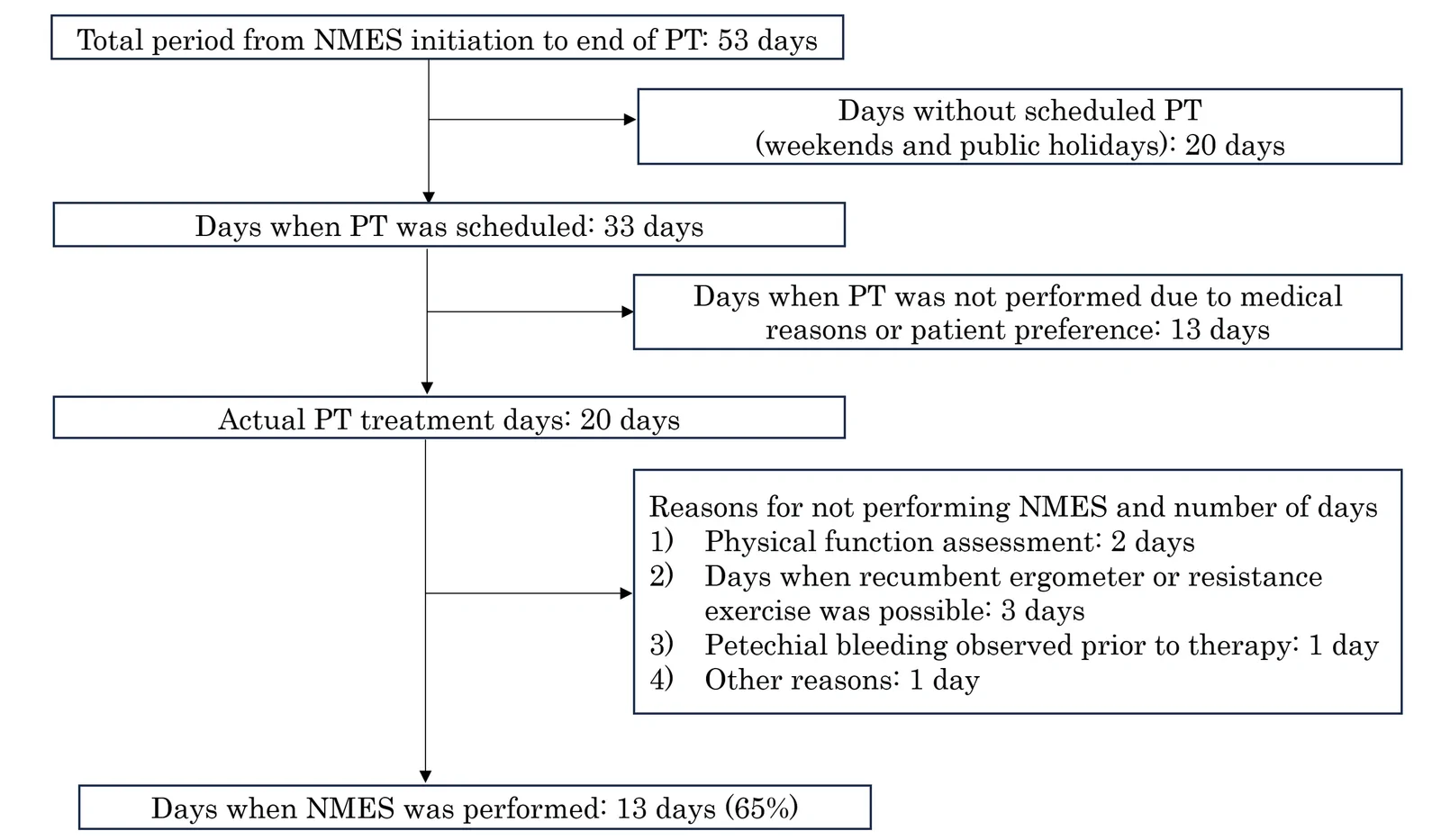
Flow of PT scheduling and NMES implementation PT, Physical Therapy; NMES; Neuromuscular electrical stimulation

The NMES intensity was set to the maximum tolerable level that did not exceed the patient's pain threshold, based on the intensity setting method reported by Nonoyama et al [17]. No transient muscle soreness, delayed-onset muscle soreness, or treatment-related fatigue occurred after the NMES sessions.

Physical outcomes

On day X+98, the results of the physical function assessment showed the following: grip strength 14.7/13.6 kg (Rt/Lt), CS5 12.2 s, 4-m gait 4.8 s (0.83 m/s), 10-m gait 10.3 s (0.97 m/s), and CC 30.5/30.0 cm (Table 1). From X+39 to X+98 days, grip strength increased by 8.7 kg on the right and 8.1 kg on the left, exceeding the previously reported minimal clinically important difference (MCID) of 5 kg [18]. CS5 decreased by 13.1 s, exceeding the previously reported minimal important difference (MID) of 1.9 s [19]. The 4-m gait speed increased by 0.35 m/s, exceeding the previously reported MCID of 0.11 m/s [20], and the 10-m gait speed increased by 0.45 m/s, exceeding the previously reported MCID of 0.10 m/s [21]. However, because these thresholds were established in patient populations other than patients with hematological malignancies, the clinical significance of these changes should be interpreted with caution. Nutritional parameters also improved: albumin from 3.0-3.9 g/dL (reference range, 4.1-5.1 g/dL [16]) and total protein from 5.3-6.7 g/dL (reference range, 6.6-8.1 g/dL [16]) (Table 2).

## Discussion

In this case of cachexia associated with B-cell ALL, NMES was applied at 20 Hz for 20 min with a duty cycle of 5 seconds on and 2 seconds off as an alternative therapy. Aerobic and resistance exercise were not feasible due to severe fatigue and thrombocytopenia. No adverse events, including pain or bleeding, were observed during the NMES sessions.

The safety and appropriate indications for NMES in patients with hematologic malignancies and severe thrombocytopenia have not been fully established. NMES has been associated with adverse events, such as pain and discomfort [22]. Bleeding associated with thrombocytopenia is a notable adverse event of exercise therapy in patients with hematological malignancies [23]. Plt count influences exercise therapy intensity. In this case, because the Plt count was 19,000/µL (reference range, 158,000-348,000/µL [16]), only gentle exercises such as sitting, standing, and stretching were considered appropriate. Therefore, NMES was introduced as an alternative approach to provide muscle stimulation when conventional exercise could not be adequately performed. NMES was safely applied at 20 Hz for 20 minutes with a duty cycle of 5 seconds on and 2 seconds off at the maximum tolerable intensity that produced visible muscle contractions. NMES was feasible on more treatment days than resistance or aerobic exercises despite severe fatigue. In this case, NMES was performed safely under these stimulation parameters despite a Plt count as low as 19,000/µL (reference range, 158,000-348,000/µL [16]).

This case report has several limitations. First, as this is a single case report, it is difficult to generalize the safety and feasibility of NMES in patients with severe thrombocytopenia. Second, the stimulation intensity of NMES was not recorded using quantitative measures such as milliamperes (mA), which limits the reproducibility of the intervention. The effects of NMES on physical function remain unknown. The improvement in grip strength may have been influenced by multiple factors, including overall physical recovery, physical therapy interventions other than NMES, and improved nutritional status. Therefore, the specific contribution of NMES to grip strength improvement could not be determined. Although no bleeding was observed at a Plt count of 19,000/μL (reference range, 158,000-348,000/µL [16]), this result may depend on the specific stimulation intensity and electrode placement used. Further research is necessary to elucidate the incidence of adverse events associated with NMES in patients with hematological malignancies and cachexia. In the future, large-scale studies will be required to establish standardized interventions and to evaluate the long-term effects of NMES on cachexia and clinical outcomes in this patient population.

## Conclusions

In this case, a patient with B-cell ALL was able to undergo NMES without any adverse events, despite severe thrombocytopenia (Plt count: 19,000/μL; reference range, 158,000-348,000/µL. This case demonstrates that NMES was feasible and well tolerated under the reported stimulation parameters in one patient with B-cell ALL; confirmation through prospective studies with larger samples is required.
